# Force-induced increased osteogenesis enables accelerated orthodontic tooth movement in ovariectomized rats

**DOI:** 10.1038/s41598-017-04422-0

**Published:** 2017-06-20

**Authors:** Qinggang Dai, Siru Zhou, Peng Zhang, Xuhui Ma, Nayong Ha, Xiao Yang, Zhifeng Yu, Bing Fang, Lingyong Jiang

**Affiliations:** 1 Center of Craniofacial Orthodontics, Department of Oral and Cranio-maxillofacial Surgery, Ninth People’s Hospital, Shanghai Jiaotong University School of Medicine, Shanghai Key Laboratory of Stomatology, Shanghai, China; 2 Department of Pediatric Dentistry, Ninth People’s Hospital, Shanghai Jiaotong University School of Medicine, Shanghai Key Laboratory of Stomatology, Shanghai, China; 3 The 2nd dental center, Ninth People’s Hospital, Shanghai Jiaotong University School of Medicine, Shanghai, China; 4 Department of Oral and Maxillofacial-Head and Neck Oncology, Ninth People’s Hospital, Shanghai Jiaotong University School of Medicine, Shanghai Key Laboratory of Stomatology, Shanghai, China; 5 Shanghai Key Laboratory of Orthopedic Implants, Department of Orthopedic Surgery, Shanghai Ninth People’s Hospital, Shanghai Jiaotong University School of Medicine, Shanghai, China

## Abstract

As the number of elderly orthodontic patients increases, the impact of postmenopausal osteoporosis on orthodontic tooth movement (OTM) has attracted a great deal of attention because OTM relies on alveolar bone remodeling. The question of whether OTM causes subsequent alveolar bone loss and is harmful to alveolar bone health under osteoporotic conditions remains to be answered. The present study aimed to clarify the influences of OTM on alveolar bone in osteoporotic rats. OTM was accelerated in ovariectomized (OVX) rats as a result of increased bone resorption in the pressure area. At the same time, anabolic bone formation was promoted in the tension area during OTM in OVX rats. Micro-CT analysis of alveolar bone revealed a decrease in BMD, BV/TV and Tb.Th. in the OTM group compared with that in non-OTM rats on day 21 of OTM, suggesting that OTM caused alveolar bone loss in OVX rats during OTM. However, the OTM-induced bone loss could be recovered 3 months after OTM in OVX rats. Thus, our findings suggest that increased osteogenesis may compensate for the increased bone resorption during and after OTM and enable effective accelerated OTM in OVX rats.

## Introduction

With increased life-span, there is a concomitant increase in the number of elderly patients needing to undergo orthodontic treatment in preparation for prosthodontic treatment or to prevent periodontal diseases^1, 2^. Because of the high incidence of osteoporosis in these aged patients, the effect of osteoporosis on orthodontic tooth movement (OTM) is receiving more attention^3–5^. Osteoporosis is characterized by low bone mass and a reduction in bone trabecular number^6, 7^. Postmenopausal osteoporosis is an ovarian estrogen-deficiency condition which causes excessive bone remodeling, “increasing bone resorption disproportionately more than bone formation”^8^. Ovariectomized (OVX) rodents, an animal model widely used to imitate postmenopausal osteoporosis^7, 9–11^, display loss of bone mass and increased bone turnover in the alveolar bone^12, 13^. OTM is a process that relies on alveolar bone remodeling in the pressure area and bone resorption and formation in the tension area. Hence, it is reasonable to believe that postmenopausal osteoporosis will affect tooth movement^14–16^.

It has been reported that estrogen deficiency accelerates OTM in OVX rats^2, 5, 17–20^. Arslan *et al*. observed an increase in the number of osteoclasts in the periodontal tissue during accelerated OTM in OVX rats^17^. Tan *et al*. suggested that the increased rate of OTM in OVX rats was related to differential expression of OPG and RANKL, which are the key factors regulating osteoclast differentiation^18^. Thus, it seems that increased bone resorption caused by estrogen deficiency is the main cause of accelerated OTM in OVX rats. However, it is not clear whether estrogen deficiency results in loss of bone mass and damage to alveolar bone during OTM, which is the key factor in the safe application of OTM. In the present study, we aimed to clarify the influences of OTM on alveolar bone in osteoporotic rats by analyzing the physical and metabolic changes of alveolar bone during and after OTM in OVX rats.

## Materials and Methods

### Animals

Ninety-six 6-week-old non-pregnant female Sprague-Dawley rats (approximate weight 180–200 g) were utilized in the present study. All rats were housed under standard conditions: room temperature 24 ± 2 °C, humidity 50–55%, standardized laboratory pellet food and water available *ad libitum*. The experimental procedures (Appendix Fig. 1A) were approved by the ethics committee of Shanghai Ninth People’s Hospital, Shanghai Jiao Tong University School of Medicine.

### Ovariectomy

Fifty-four rats were randomly selected for bilateral ovariectomy, and the other forty-two rats were subjected to sham-ovariectomy (Sham). The surgical procedures were performed according to the FDA guidelines (Appendix Fig. 1B)^21^. All rats were housed under the afore-mentioned conditions for 3 months^22^.

### Orthodontic tooth movement

Three months after surgery, OTM was performed following the method described in previous studies^23, 24^. Generally, the maxillary left molars were moved mesially by closed-coil springs (3 M Unitek, Monrovia, CA, USA) ligatured by a 0.2 mm steel ligature between the maxillary first molar and the incisors, which could generate force of a magnitude of 50 g (Appendix Fig. 1F). To enhance the attachment of the ligature to the incisors, 0.5-mm grooves were drilled at the tooth cervix of the incisors and self-curing resin was used. Six rats in each group were euthanized by overdose of chloral hydrate at the appropriate time points (day 0, 1, 3, 7, 15 and 21), and the samples were used for histological study. The amount of tooth movement was defined as the distance between the most mesial point of the maxillary second molar and the most distal point of the first molar measured by an electronic caliper. Each distance was measured twice by the same observer, and the mean was used.

The uteri and femora from rats sacrificed on day 0 were used for hematoxylin & eosin (H-E) staining to confirm OVX status and for dual energy X-ray absorptiometry (DXA) scanning to determine bone mineral density (BMD).

### Histology and immunohistochemistry

Histological examination was performed as prescribed^25, 26^. Briefly, after sacrifice of the animals, maxillae were fixed with 4% paraformaldehyde for 48 hours. Samples were decalcified with 10% EDTA for 8 weeks, then embedded in paraffin. Consecutive 4-μm sections were obtained from the sagittal direction, and the sections containing the disto-palatal roots were selected. Osteoclasts were detected as wine-red multinuclear cells in sections after tartrate-resistant acid phosphatase (TRAP) staining, which was performed using an acid phosphatase leukocyte kit (Sigma, St Louis, MO, USA)^1^.

Immunohistochemical staining was performed according to a previously-published method^27^. Briefly, the samples were deparaffinized and rehydrated, then incubated in 0.2% proteinase K for 20 min at 37 °C for antigen retrieval. Next, 3% H_2_O_2_ was used to block endogenous peroxidase activity. Sections were incubated with mouse monoclonal IgG-anti runt-related transcription factor 2 (Runx2, dilution 1:200, Abcam, Cambridge, MA, USA) and osteocalcin (Ocn, dilution 1:200, Abcam) overnight at 4 °C, followed by horseradish peroxidase-conjugated secondary antibody (DAKO, Glostrup, Denmark). Samples were stained with DAB substrate (DAKO) and counterstained with hematoxylin.

The distal coronal one-third area of the disto-palatal root was selected as the tension area, while the pressure area was the mesial coronal one-third area (Appendix Fig. 1G). All histological sections were observed using a light microscope and a digital camera (Carl Zeiss, Oberkochen, Germany). All histological measurements were performed with Image-Pro Plus 6.0 (Media Cybernetics, Bethesda, MD, USA). Osteoclasts (identified by TRAP-positive staining) were counted in the pressure area, while the numbers of Runx2- and osteocalcin-positive cells were counted in the tension area.

### Sequential fluorochrome labeling and histomorphometric analysis

Twelve rats (six sham and six OVX) received intraperitoneal injection of 20 mg/kg BW calcein (CA; Sigma) on day 5, and 25 mg/kg BW alizarin red (AL; Sigma) on day 19^28, 29^. These rats were sacrificed on day 21 and the maxillae were dehydrated and embedded in polymethylmethacrylate. Samples were cut into 200 μm-thick sections along the occlusion plane with a microtome (Leica, Wetzlar, Germany). The sections of the coronal one-third were polished to a thickness of 100 µm and used to observe fluorescent labeling with a confocal laser scanning microscope (Leica). The excitation/emission wavelengths of the chelating fluorochromes used were 488/500–550 nm for CA (green) and 543/580–670 for AL (red)^28, 29^. Both mineral apposition rate (MAR) and bone formation rate (BFR/BS) were measured following a previously-described method^30^.

### Micro-CT analysis of alveolar bone

To study the microarchitectural changes in alveolar bone during OTM in OVX rats, 12 OVX rats were subjected to micro-CT scanning. Six rats were sacrificed on the 21^st^ day of OTM. The tooth movement devices of the other six rats were removed on the same day and they were sacrificed 3 months after OTM. All maxillae were scanned with a micro-CT scanner (Scanco, Switzerland) with a voxel size of 18 μm. BMD; bone volume fraction (BV/TV); trabecular thickness (Tb.Th.); trabecular number (Tb.N.); and trabecular separation (Tb.Sp.) of alveolar bone were analyzed at the inter-radicular region of the maxillary first molar following a previously-described method^13^.

### Statistical analysis

All data are presented as mean ± SD. All statistical analyses were carried out by one-way analysis of variance with SPSS, version 16.0 (SPSS Inc., Chicago, IL, USA), and values of *P* < 0.05 were considered to indicate a significant difference.

## Results

### Ovariectomy results in accelerated tooth movement with increased bone resorption in rats

**Figure 1:**
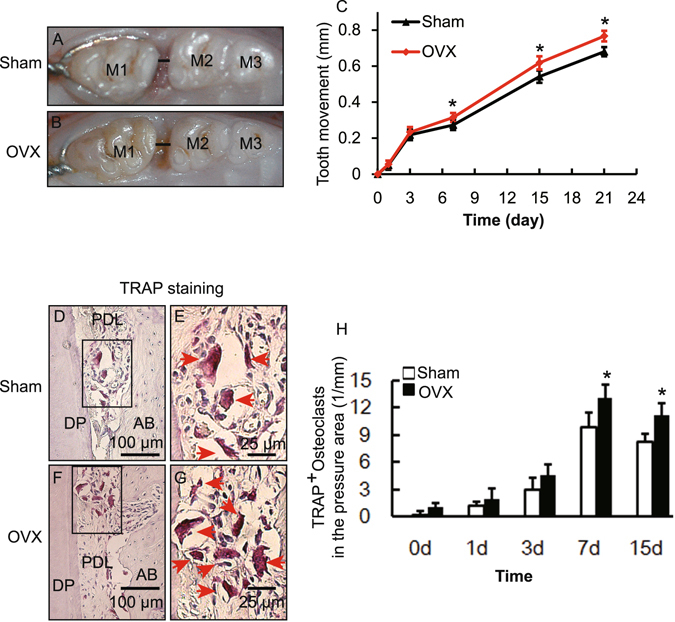
Ovariectomy increased OTM and bone resorption during OTM in OVX rats. (**A**,**B**) Representative images of tooth movement on day 21 of OTM in Sham and OVX rats. M1, the left maxillary first molar; M2, the left maxillary second molar; M3 the left maxillary third molar; the distance of OTM is indicated by a black line. (**C**) Effect of ovariectomy on the rate of OTM. Data represent means ± S.D. *P < 0.05, n = 6. (**D**–**G**) Representative images of TRAP staining in the pressure area on day 7 of OTM in Sham and OVX rats. E and G are high-magnification images of the squares in D and F respectively. AB, alveolar bone; DP, disto-palatal root; PDL, periodontal ligament; P, pressure area; T, tension area. (**H**) TRAP-positive osteoclasts in the pressure area during OTM in Sham and OVX rats. Data represent means ± S.D. *P < 0.05, n = 6.

To study the effect of osteoporosis on OTM, we constructed a rat model of osteoporosis by performing ovariectomy following the FDA’s guidelines. The success of our osteoporosis model was confirmed by atrophy of the uteri (Fig. 1C,E) and decreased BMD of the femora (Appendix Fig. 1D) 3 months after surgery in OVX rats.

Next, we performed OTM of the left maxillary molar with a widely-used model in both Sham and OVX rats. We found that both Sham and OVX rats exhibited similar tooth movement in the first 7 days (Fig. 1C). There was a phase of rapid tooth movement during the first 3 days, followed by decreased tooth movement from days 3 to 7. A linear phase of tooth movement was detected after day 7. However, tooth movement in OVX rats was increased after day 7 when compared with the corresponding Sham group (Fig. 1A–C).

Osteoclast activity (bone resorption) in the pressure area is critical to the tooth movement rate. In the present tipping model of maxillary first molar movement, the mesial coronal one-third area of the disto-palatal root was considered as the pressure area and the tension area was the distal coronal one-third area (Appendix Fig. 1F,G)^18^. To test our hypothesis that the increased OTM in OVX rats results from increased bone resorption, we determined the activity of osteoclasts in the pressure area by TRAP staining. TRAP activity increased after the application of orthodontic force in both OVX and Sham groups (Fig. 1D–G). Furthermore, the number of TRAP-positive osteoclasts was increased in comparison with the Sham group in OVX rats on days 7 and 15 (Fig. 1D–H). These results indicated that ovariectomy promoted catabolic bone resorption during the linear phase of OTM, which might result in accelerated OTM in OVX rats.

### Ovariectomy induces increased osteogenesis during OTM in rats

**Figure 2:**
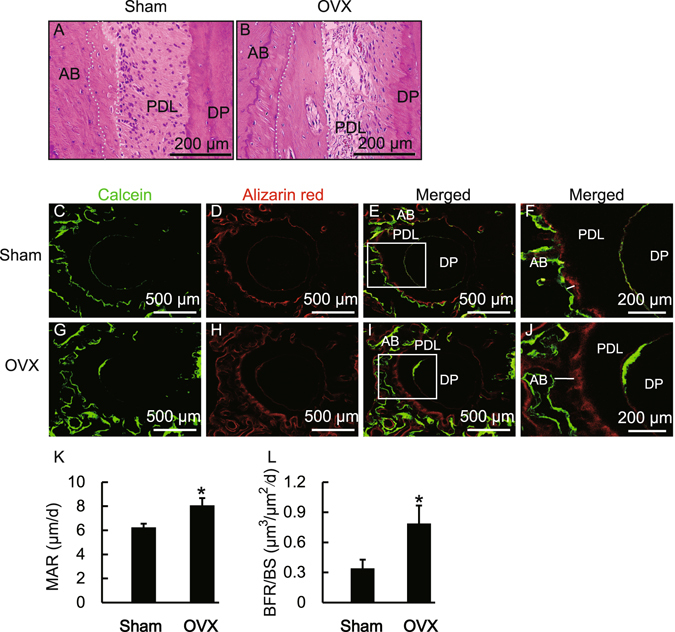
Ovariectomy-induced promotion of bone formation in the tension area during OTM in OVX rats. (**A**,**B**) H–E staining of the tension area on day 21 in Sham and OVX rats. The white dotted line indicates newly-formed bone. (**C**–**J**) Effects of OVX on bone formation rate during OTM. F and J show high magnification images of the squares in E and I respectively. The white line indicates newly-formed bone. (**K**,**L**) OVX increased the mineral apposition rate (MAR) and bone formation rate (BFR/BS) in the tension area during OTM in OVX rats. Data represent means ± S.D. *P < 0.05, n = 6.

Ovariectomy increased bone resorption during accelerated OTM in OVX rats, which may result in loss of alveolar bone mass and reduce the safety of OTM. Thus, we asked if osteogenesis would be enhanced and compensate for alveolar bone loss caused by the increased bone resorption during OTM in OVX rats. To test this, we analyzed the amount of newly-formed bone in the tension area on day 21 by H-E staining. As shown in Fig. 2A,B, new bone was deposited along the alveolar bone edge in the tension area in both OVX and Sham rats. Interestingly, there was more newly-deposited bone in the OVX rats in comparison with the Sham rats, which suggested that ovariectomy promoted osteogenesis during OTM.

To further determine the effect of estrogen-deficiency on anabolic metabolism during OTM in OVX rats, we used fluorescent labeling of CA (green) and AL (red) to analyze the area of bone formation. As shown in Appendix Fig. 2, in the non-OTM group of both Sham and OVX rats, a weak but evenly-distributed fluorescent signal was visible along the alveolar bone around the disto-palatal root. However, in the OTM group, the fluorescent labeling on the distal side of the disto-palatal root (tension area) was dramatically strong, while the fluorescent signal on the mesial side (pressure area) was weaker (Fig. 2C–J). These results confirmed that new bone was deposited in the tension area during OTM. We further determined the rate of bone formation in the tension area by histomorphometric analysis. As shown in Fig. 2K,L, both mineral apposition rate and bone formation rate increased in the OVX rats in comparison with those in the Sham group. These results indicate that ovariectomy promotes anabolic bone formation during OTM in OVX rats.

**Figure 3:**
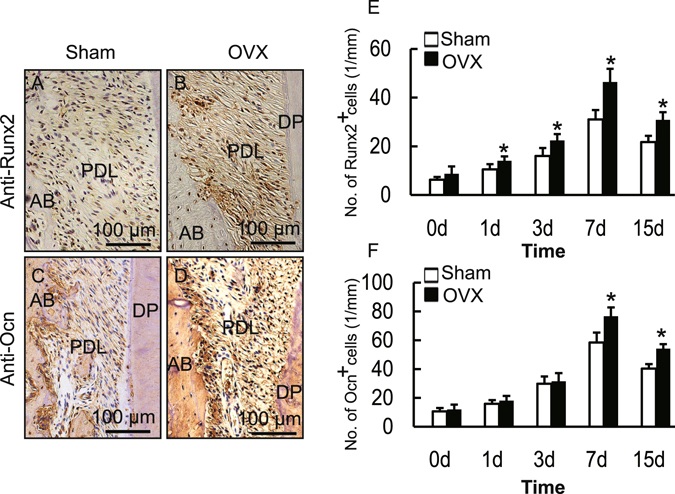
Ovariectomy promoted the expression of Runx2 and osteocalcin in the tension area produced by OTM in OVX rats. (**A**–**D**) Immunohistochemical staining of Runx2 or osteocalcin (Ocn) in the tension area on day 7 of OTM in Sham and OVX rats. (**E**,**F**) Number of Runx2- or osteocalcin-positive cells in the tension area during OTM. Data represent means ± S.D. *P < 0.05, n = 6.

To understand the molecular mechanism underlying the changes in bone formation during OTM in OVX rats, we analyzed bone-forming activity by investigating the presence of osteoblasts. This was carried out by examining the expression level of Runx2 and osteocalcin in the tension area. As shown in Fig. 3, the numbers of both Runx2- and osteocalcin-positive cells increased in the OVX rats in comparison with those in the Sham group, which indicated that both immature and mature osteoblasts increased in OVX rats.

### Alveolar bone recovery after OTM in OVX rats

**Figure 4:**
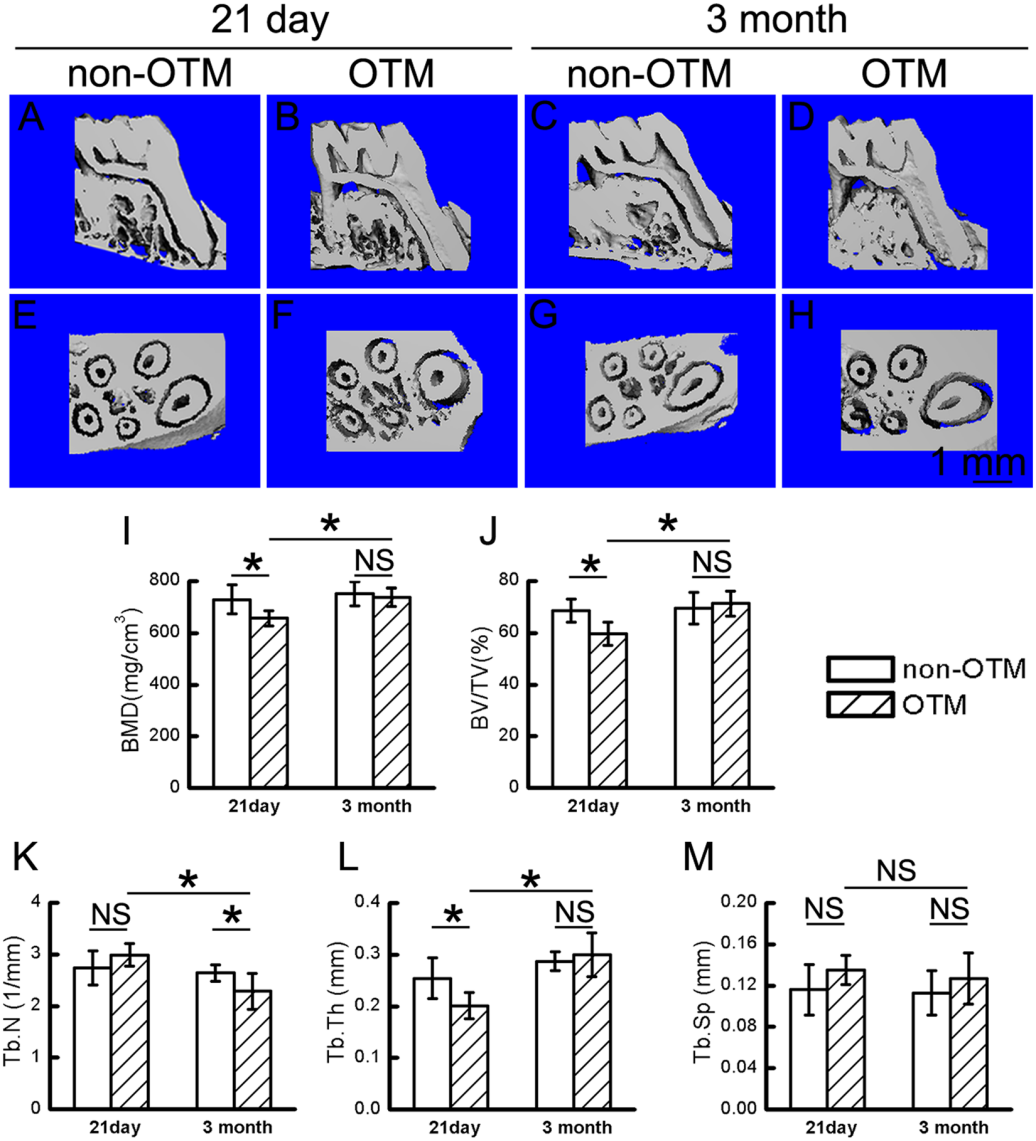
Alveolar bone recovery after OTM in OVX rats. (**A**–**H**) Three-dimensional reconstruction of alveolar bone in the Non-OTM and OTM groups on day 21 of OTM (21 day) and 3 months after OTM (3 month) in OVX rats. (**I**–**M**) Analysis of microstructural parameters of alveolar bone during and after OTM in OVX rats. BMD, bone mineral density; BV/TV, bone volume fraction; Tb.Th., trabecular thickness; Tb.N., trabecular number; Tb.Sp., trabecular separation. Data represent means ± S.D. *P < 0.05, NS *P* ≥ 0.05, n = 6.

Our aforementioned data demonstrate that ovariectomy promoted both catabolic and anabolic metabolism during OTM in OVX rats; however, whether the elevated bone formation can compensate for the increased bone resorption and thus prevent bone loss during OTM remains ambiguous. To clarify this, we analyzed the alveolar bone mass and microarchitectural changes in OTM and Non-OTM groups on day 21 of OTM in the OVX rats by micro-CT. The alveolar bone of the right maxillary first molar was considered as the control group of OTM (Non-OTM). Three-dimensional reconstruction showed a porous structure with fewer bone trabeculae in the alveolar bone of the OTM group on day 21 of OTM when compared with the non-OTM group (Fig. 4A,B,E,F). Microstructure parameter analysis showed decreases of 15%, 16%, and 24%, respectively, in BMD, BV/TV and Tb.Th. in the OTM group compared with the Non-OTM group in OVX rats (Fig. 4I,J,L). There was no difference in Tb.N. or Tb.Sp. between the OTM group and the Non-OTM group on day 21 of OTM (Fig. 4K,M). These results suggested that there was a moderate alveolar bone loss during OTM in OVX rats.

Bone remodeling is a dynamic cycle of bone formation, mineralization, and bone resorption. Although there was a loss of alveolar bone during OTM in OVX rats, whether the alveolar bone loss could be recovered after OTM is more important for the safety of OTM under osteoporotic conditions. To explore this, we removed the OTM devices on day 21 of OTM in OVX rats and analyzed the changes in alveolar bone 3 months later by micro-CT. First, we compared the alveolar bone in the OTM group on day 21 and 3 months after OTM. As shown in Fig. 4B,D,F,H, the alveolar bone in the OTM group at 3 months after OTM showed more compact bone trabeculae in comparison to the bone on day 21 of OTM. BMD, BV/TV and Tb.Th. in the OTM group 3 months after OTM was increased compared to the OTM group on day 21 of OTM (Fig. 4I,J,L), while Tb.N. in the OTM group 3 months after OTM was lower than that on day 21 of OTM. Furthermore, we compared alveolar bone between the OTM and Non-OTM group 3 months after OTM in OVX rats. We found that microstructural parameters of alveolar bone, with the exception of Tb.N., were comparable between the OTM group and Non-OTM group 3 months after OTM in OVX rats. All these data indicated that alveolar bone loss during OTM could be recovered after OTM in OVX rats.

## Discussion

OTM occurs through alveolar bone remodeling in response to mechanical stimuli, and predominantly consists of bone formation in the tension area and bone resorption in the pressure area^15, 31^. It has been demonstrated that a combination of systemic hormones and local factors can affect tooth movement^32, 33^. With the increasing number of elderly orthodontic patients, the influence of osteoporosis on OTM has attracted great attention in both clinical and basic studies^3, 4, 34^. The previous studies mostly focused on the influences of osteoporosis on the rate of tooth movement, but the safety of OTM in patients with osteoporosis remains obscure and the underlying mechanism is also unclear. In the present study, OVX rats were used to mimic the remodeling of periodontal tissue during OTM under osteoporotic conditions. We found that the resulting estrogen deficiency promoted alveolar osteogenesis which may facilitate accelerated tooth movement in OVX rats.

Our previous study showed that estrogen deficiency results not only in deterioration of bone microstructure but also in increased bone resorption of alveolar bone^13^, which may cause alteration of bone remodeling during OTM. In our present study, we found that OTM displayed a similar pattern in both Sham and OVX rats, which fitted the 3-phase pattern of tooth movement^2, 35, 36^. Rapid tooth movement was observed at the beginning, with a high rate of movement during the first 3 days caused by the mechanical deformation of the periodontal tissues, followed by a lag phase from days 3 to 7. The subsequent linear phase from day 7 onward should be accompanied by balanced bone formation and bone resorption. There was no difference in the rate of OTM between Sham and OVX rats in the initial or lag stages of tooth movement, but the OTM rate was increased during the linear phase in OVX rats, which suggested that estrogen-deficiency accelerates OTM via OVX-induced changes in bone remodeling.

Alveolar remodeling during OTM includes bone resorption (by osteoclasts) in the pressure area and bone formation (by osteoblasts) in the tension area^18, 31^. In our OTM model, the appliance produced a continuous horizontal force along the occlusal plane and induced a tipping movement of the first molar. This tipping movement resulted in tension in the distal coronal one-third area of the disto-palatal root and pressure in the mesial coronal one-third area^18^. Osteoclast activity in the pressure area is the main restrictive factor affecting the rate of OTM in the linear phase. We found that bone resorption increased in the pressure area during the linear phase of OTM in OVX rats, indicated by increased TRAP activity. These data indicated that OVX-induced elevated bone resorption resulted in accelerated OTM during the linear phase in OVX rats.

The stability and safety of OTM is based on the balance of catabolic bone resorption and anabolic bone formation. Ovariectomy induced increased bone resorption and accelerated OTM in OVX rats, which may exaggerate the negative balance of bone metabolism caused by estrogen-deficiency and result in consequent alveolar bone loss, unless osteogenesis can also increase and maintain the balance with bone resorption. In our present study, we found that bone formation in the tension area indeed increased in OVX rats, determined by increased MAR, BFR and osteoblast number. These results indicated that bone formation increased during OTM in OVX rats, but whether the promoted alveolar osteogenesis would be able to compensate for the increased bone resorption and maintain the balance of bone metabolism is not clear. To investigate this we analyzed microstructural changes of the alveolar bone after 21 days of OTM in OVX rats.

On the 21^st^ day of OTM in OVX rats, alveolar bone of the OTM group showed a porotic structure compared with the non-OTM group, indicated by decreased BMD, BV/TV and Tb.Th. These results suggested that OTM may cause alveolar bone loss in OVX rats. What should be taken into account is that bone formation and mineralization lag behind bone resorption during bone remodeling, which suggests that whether alveolar can be recovered during the period after OTM is critical for the safety of this procedure. It is of interest that the loss of alveolar bone during OTM in OVX rats was recovered 3 months after OTM, indicated by increased BMD, BV/TV and Tb.Th. of the OTM group 3 months after OTM in comparison with that on day 21 of OTM. These results suggest that although OTM may cause temporary alveolar bone loss during OTM in OVX rats, bone structure would recover after OTM, which may be essential for the safety of OTM in patients with osteoporosis.

In summary, we found that the rate of OTM was increased in OVX rats, which may be related to elevated bone resorption. Simultaneously, OVX-induced promotion of osteogenesis may compensate for the increased bone resorption to some degree during and after OTM and enable the stability and safety of orthodontic treatment under osteoporotic conditions. Our present study provides evidence for the safety of adult orthodontic therapy, even for adults with osteoporosis, although more clinical data will be needed in the future.

## Electronic supplementary material

Appendix
