# Supplementary material for: Force-induced increased osteogenesis enables accelerated orthodontic tooth movement in ovariectomized rats
Source: Sci Rep. 2017 Jun 20;7:3906. doi: 10.1038/s41598-017-04422-0 (PMC5478594; doi:10.1038/s41598-017-04422-0)
Supplement: Supplementary file 1 — Appendix [file 41598_2017_4422_MOESM1_ESM.pdf]

Force-induced increased osteogenesis enables accelerated orthodontic tooth movement in ovariectomized rats

Qinggang Dai, Siru Zhou, Peng Zhang, Xuhui Ma, Nayong Ha, Xiao Yang, Zhifeng Yu, Bing Fang, Lingyong Jiang

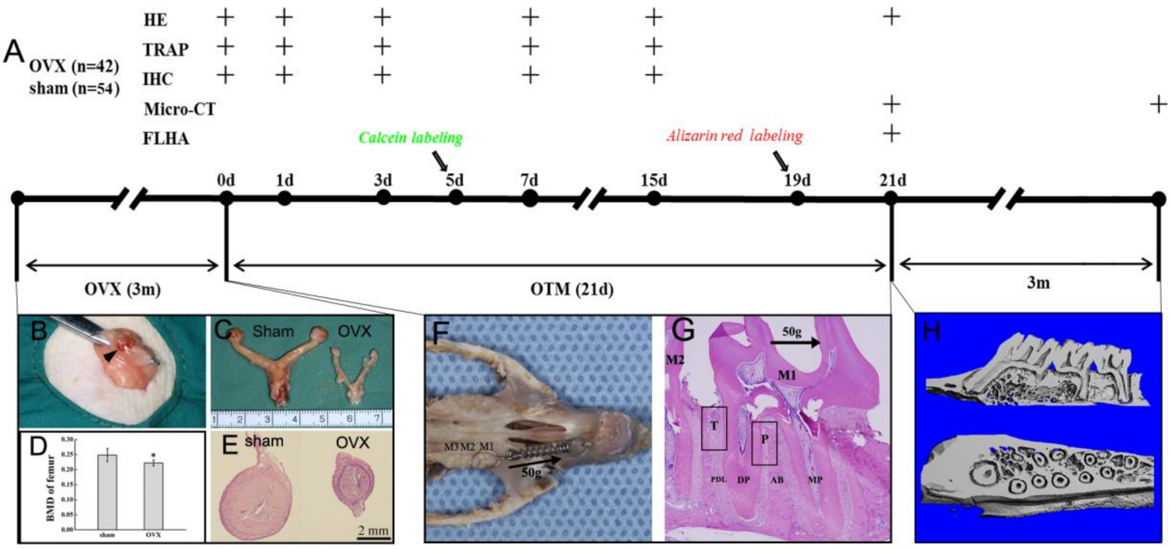

Appendix Figure 1 Time-schedule and region of interesting.

(A) the time schedule of the present study. HE, HE staining; TRAP, tartrate-resistant acid phosphatase staining; IHC, immunohistochemistry; FLHA, fluorochrome labeling histomorphometrical analysis.

(B) Surgery of ovariectomy. Arrow head indicated ovary

(C and E) Ovariectomy induced uteri atrophy 3 months after surgery.

(D) Ovariectomy deceased bone mineral density of femur measured by dual energy X-ray. Data represent means ± s.d.* *P*<0.05, n=6.

(F) The maxillary first molars were mesially moved by closed-coil springs. M1, the left maxillary first molar; M2, the left maxillary second molar; M3, the left maxillary third molar; arrows indicated the direction of OTM.

(G) Region of interesting during OTM. The distal coronal one-third area of the disto-palatal root was the tension area (T) and the pressure area (P) was the mesial coronal one-third area.; AB, alveolar bone; DP, disto-palatal root; MP, mesio-palatal root.

(H) Micro-CT images of alveolar bone.

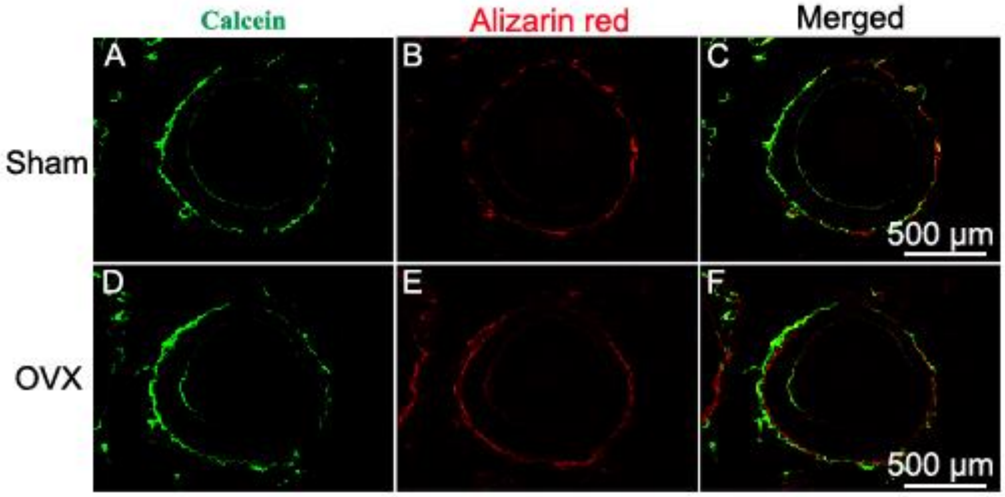

Appendix Figure 2 Fluorochrome labeling of alveolar bone formation in Non-OTM group in Sham and OVX rats.

(A-C) Representative images of fluorochrome labeling of alveolar bone around the disto-palatal root in Non-OTM group in Sham rats.

(D-F) Representative images of fluorochrome labeling of alveolar bone around the disto-palatal root in Non-OTM group in OVX rats.
